# Results of a pilot risk-based lung cancer screening study: outcomes and comparisons to a Medicare eligible cohort

**DOI:** 10.1007/s12672-023-00773-5

**Published:** 2023-08-29

**Authors:** Erin A. Hirsch, Melissa L. New, Stephanie L. Brown, Stephen P. Malkoski

**Affiliations:** 1 Division of Medical Oncology, University of Colorado Anschutz Medical Campus, 12700 E 19th Ave, Aurora, CO 80045 USA; 2 Pulmonary Section, Rocky Mountain Regional VA Medical Center, 1700 N. Wheeling Street, Aurora, CO 80045 USA; 3 Division of Pulmonary Sciences and Critical Care Medicine, University of Colorado Anschutz Medical Campus, 12700 E 19th Ave, Mail Stop C272, Aurora, CO 80045 USA; 4 University of Colorado Hospital, UCHealth Denver Metro, Aurora, CO 80045 USA; 5 Department of Medicine, University of Washington, WWAMI – Spokane, 502 E Boone Ave, Spokane, WA 99258 USA; 6 Sound Critical Care, Sacred Heart Medical Center, 101 W. 8th Avenue, Spokane, WA 99204 USA

**Keywords:** Cancer Screening, Lung cancer screening, Risk-based screening, Lung cancer

## Abstract

**Purpose:**

Risk-based lung cancer screening holds potential to detect more cancers and avert more cancer deaths than screening based on age and smoking history alone, but has not been widely assessed or implemented in the United States. The purpose of this study was to prospectively identify patients for lung cancer screening based on lung cancer risk using the PLCO_m2012_ model and to compare characteristics, risk profiles, and screening outcomes to a traditionally eligible screening cohort.

**Methods:**

Participants who had a 6 year lung cancer risk score ≥ 1.5% calculated by the PLCO_m2012_ model and were ineligible for screening under 2015 Medicare guidelines were recruited from a lung cancer screening clinic. After informed consent, participants completed shared decision-making counseling and underwent a low-dose CT (LDCT). Characteristics and screening outcomes of the study population were compared to the traditionally eligible Medicare cohort with Fisher’s Exact, t-tests, or Brown Mood tests, as appropriate.

**Results:**

From August 2016 to July 2019, the study completed 48 baseline LDCTs. 10% of LDCTs recommended further pulmonary nodule evaluation (Lung-RADs 3 or 4) with two early-stage lung cancers diagnosed in individuals that had quit smoking > 15 years prior. The study population was approximately 5 years older (p = 0.001) and had lower pack years (p = 0.002) than the Medicare cohort.

**Conclusion:**

Prospective application of risk-based screening identifies screening candidates who are similar to a traditionally eligible Medicare cohort and future research should focus on the impact of risk calculators on lung cancer outcomes and optimal usability in clinical environments.

This study was retrospectively registered on clinicaltrials.gov (NCT03683940) on 09/25/2018.

## Purpose

Large clinical trials have shown that lung cancer screening (LCS) with low dose computed tomography (LDCT) reduces lung cancer specific mortality by at least 20% in high-risk individuals [1, 2], and guidelines that drive insurance coverage are based on age and cigarette smoking history [3–5]. In 2015, the Centers for Medicare and Medicaid Services (CMS) recommended LCS in individuals 55–77 years old with a ≥ 30 pack year smoking history who currently smoke or have quit within the past 15 years [3]. Based on modeling data, the US Preventive Services Task Force (USPSTF) recommends screening through age 80 [4]. Recently, CMS and USPSTF expanded LCS guidelines by lowering age eligibility to 50 and tobacco exposure to 20 pack years [4, 5].

Risk-based screening defines screening eligibility based on individual risk factors and may detect more cancers and avert more cancer deaths [6–8]. There are several risk prediction calculators available, but the PLCO_m2012_ model [7] is one of the more accurate at identifying individuals with a history of cigarette smoking for screening and is based on eleven variables (age, race, education,, body mass index, presence of COPD/emphysema, personal history of cancer, family history of lung cancer, smoking status (current or former), cigarettes smoked per day, years smoked, years since quitting smoking) [8]. Early assessments of risk-based LCS have predominantly been based on modeling data or applied to screening cohorts retrospectively [8–10], and have more recently been tested prospectively in international studies [11, 12]. However, risk-based screening has not been widely assessed or implemented in the United States. The aim of this study was to prospectively identify and screen patients for lung cancer based on lung cancer risk using the PLCO_m2012_ model and to compare characteristics, risk profiles, LDCT outcomes, and lung cancer diagnoses to a LCS cohort that met standard screening eligibility. Some of the results of these studies have been previously reported in the form of an abstract, [13] this manuscript provides additional information on comparing characteristics and screening outcomes with the Medicare-eligible cohort.

## Methods

Eligible individuals were identified from primary care referrals to the University of Colorado Hospital LCS clinic between August 2016 and July 2019. In our LCS program, all referrals are screened by the program nurse navigator to determine screening eligibility per CMS guidelines. Study enrollment was offered to individuals 40–82 years of age with a 6-year risk score of ≥ 1.5% if < 77 years old or > 4% if 78–82 years old (calculated by the PLCO_m2012_ model) *who did not meet 2015 CMS screening criteria*. Individuals were excluded if (1) they were eligible for screening based on 2015 CMS guidelines, (2) had a chest CT within the past year, (3) had symptoms consistent with lung cancer, (4) were unwilling to be treated for lung cancer, or (5) they had life expectancy of < 6 months. This study conforms to standards of the Declaration of Helsinki and was approved by the Colorado Multiple Institutional Review Board (15-1694).

After informed consent, study participants completed the LCS process by engaging in shared design-making with a LCS clinician, were offered tobacco cessation by a certified tobacco treatment specialist if currently smoking, underwent a LDCT read by board-certified thoracic radiologists, and received follow-up and additional referrals from the LCS clinic. Study participants could return annually to the LCS clinic for an updated assessment of risk and could undergo up to three annual LDCTs as part of the study.

Demographic and clinical data, including smoking history, 6-year risk score, LDCT outcomes, and eligibility were collected. Descriptive statistics and univariate differences between the study population and the traditionally eligible CMS LCS cohort, screened between July 2014 and December 2018 (described in [14]), were compared with Fisher’s Exact tests for categorical variables and t-tests or Brown-Mood tests for normally and non-normally continuous variables, respectively. Data were analyzed with SAS 9.4 software (SAS Institute Inc., Cary, NC).

## Results

**Fig. 1:**
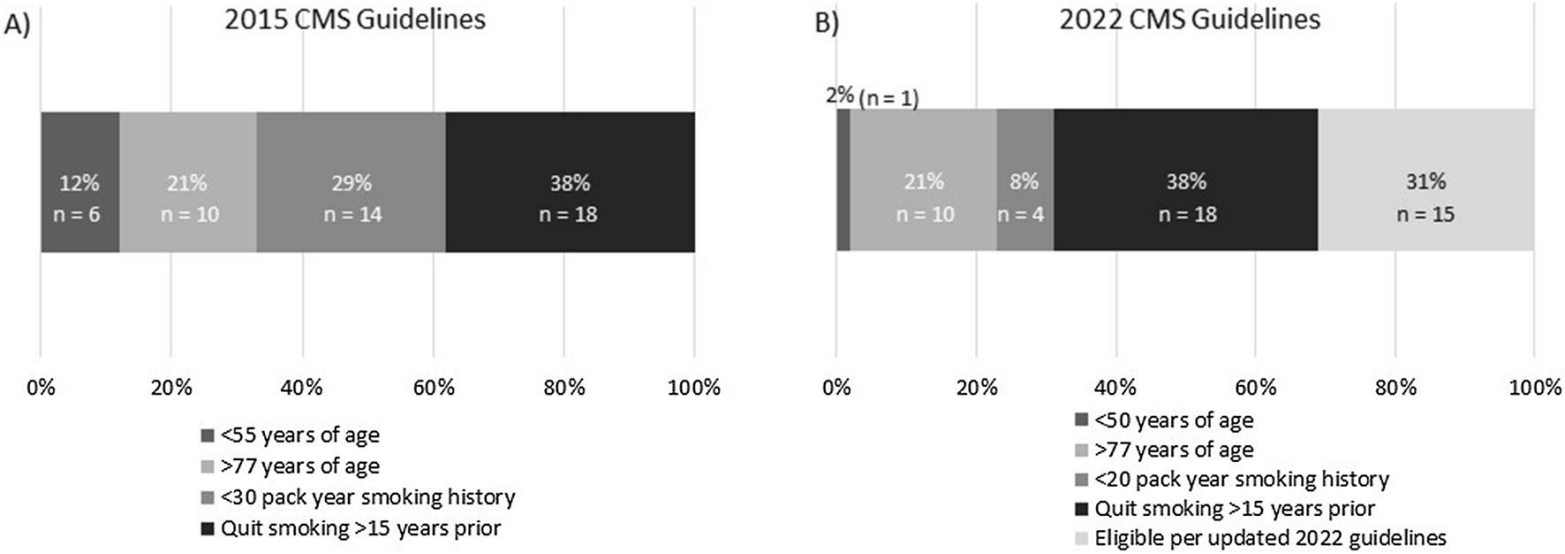
Reasons study individuals were ineligible for lung cancer screening per CMS guidelines. **A** 2015 CMS guidelines **B** 2022 CMS guidelines. Two study participants were ineligible by 2015 CMS guidelines for both being > 77 years of age and having less than a 30 pack year smoking history. These individuals are included in the > 77 years of age category because they remain ineligible for screening by the 2022 CMS guidelines due to the upper age limit criterion

Between August 2016 and July 2019, there were 66 LDCTs performed on 48 participants. Two-thirds (n = 32) of the study population had one LDCT, 14 participants had two LDCTs, and two participants completed three LDCTs. Compared to the traditionally eligible LCS cohort, the study population was older and had less cigarette smoking exposure (Table 1). LDCT outcomes were similar between the study population and the traditional screening cohort. Two study participants were diagnosed with lung cancer, a stage 1A adenocarcinoma in a white female with a 3.3% risk score and a carcinoid in a white male with a risk score of 7.5%. Both individuals had quit smoking > 15 years prior to their baseline (first study) LDCT. Figure 1A depicts the reasons that study individuals were ineligible for screening by 2015 Medicare guidelines. Under the 2022 Medicare guidelines, 69% of the study population remains ineligible for LCS, predominantly because of time from smoking cessation (Fig. 1B). Both individuals diagnosed with lung cancer remain ineligible for screening under 2022 Medicare guidelines.

**Table 1:** Characteristics of lung cancer screening populations and LCS outcomes

	Study population n = 48	Traditional 2015 CMS cohort n = 624^a^	p-value
Sex, Male (n, %)	25 (52)	355 (57)	0.55
Race, non-hispanic white (n, %)	40 (83)	521 (85)	0.84
Age at baseline scan (± SD)	69.0 (± 9.0)	64.3 (± 6.0)	0.001
Currently smoke cigarettes (n, %)	23 (48)	321 (51)	0.66
Median pack years (IQR)	33.4 (23.5–45.0)	41.0 (36.0–54.0)	0.002
Median 6 year risk score (IQR) (%)	3.4 (2.3–5.7)	3.2 (1.9–5.7)	0.55
Baseline LDCT lung-RADs result (n, %)			
1	22 (46)	355 (57)	0.39
2	21 (44)	189 (30)	
3	2 (4)	41 (7)	
4A	2 (4)	15 (2)	
4B	0 (0)	4 (1)	
4X	1 (2)	20 (3)	
Significant incidental finding on baseline LDCT (n, %)	17 (35)	160 (26)	0.17
Lung cancer diagnosis (n, %)	2 (4)	13 (2)	0.29

^a^The traditional 2015 CMS cohort includes all individuals screened for lung cancer at the University of Colorado Hospital between July 2014 and December 2018. Data are presented as count (%), mean ± SD, or median (IQR). The 6 year risk score was calculated with the PLCO_m2012_ model based on eleven variables (age, race, education, body mass index, presence of COPD/emphysema, personal history of cancer, family history of lung cancer, smoking status (current or former), cigarettes smoked per day, years smoked, years since quitting smoking). The study population was about 5 years older (p = 0.001) and had less tobacco exposure (p = 0.002)

## Discussion

Findings from this pilot study demonstrate that early-stage lung cancers can be detected in asymptomatic individuals who are prospectively selected by risk calculators and fall outside 2015 CMS guidelines. This is consistent with other studies reporting that the PLCO_m2012_ model is superior and more sensitive than 2013 and 2021 USPSTF guidelines for LCS selection [11, 15]. We also found that the PLCO_m2012_ model selects high-risk individuals for LCS that are comparable to a 2015 CMS eligible cohort in risk profile, LDCT outcome, and lung cancer diagnoses. Not surprisingly, the study population is somewhat older and has a lower cigarette smoking history than a 2015 CMS guideline LCS cohort.

Recently CMS and USPSTF expanded LCS eligibility, in part, to narrow racial and ethnic disparities [4, 5, 16]. Only 31% of our study population gains LCS eligibility under these guidelines, including 50% of racial/ethnic minority and 25% of non-Hispanic White individuals (data not show). Since clinical trials have not assessed LDCT in individuals who quit smoking > 15 years prior, CMS guidelines state there is insufficient evidence to inform screening in this population [5]. In this study, both lung cancers were diagnosed in participants who had quit smoking > 15 years before the baseline LDCT. Clearly this is an area that merits further investigation.

Although prospective, this study is primarily limited by its small size and low representation of racial and ethnic minorities; our observations need to be replicated in larger high-risk populations. We also do not have resources available to follow long-term adherence and lung cancer outcomes in this high-risk group. Both the National Lung Screening Trial [1] and Dutch-Belgian Lung Cancer Screening Trial [2] diagnosed more early-stage lung cancers on follow-up rounds of screening than on the baseline scan, indicating the importance of annual screening among eligible individuals. It may be possible that future lung cancers will be diagnosed in individuals that participated in this study. Recently, the use of risk calculators to select high-risk individuals for LCS was highlighted as a priority research area as the benefits and effect on outcomes compared to eligibility based on age and smoking history remain unclear [16]. In conclusion, prospective application of risk-based screening identifies screening candidates who are similar to a traditionally eligible Medicare cohort and future research should focus on the impact of risk calculators on lung cancer outcomes and optimal usability in clinical environments.

## Data Availability

The datasets generated during and/or analyzed during the current study are available from the corresponding author on reasonable request.

## Abbreviations

CMS: Centers for Medicare and Medicaid Services
LDCT: Low dose computed tomography
LCS: Lung cancer screening
USPSTF: United States preventive services task force
